# Community acquired Panton-Valentine Leukocidin (PVL) positive Methicilin Resistant Staphylococcal aureus cerebral abscess in an 11-month old boy: a case study

**DOI:** 10.1186/1756-0500-7-862

**Published:** 2014-12-01

**Authors:** Wilbroad Mutale, Keya M Sahay, John Hartley, Dominic Thompson, Didi Ratnasinghe, Lee Hudson, Eleanor Hulse, Greg Fellows

**Affiliations:** London School of Hygiene and Tropical Medicine, Clinical Research Unit, London, UK; Great Ormond Street Children’s Hospital, London, UK; Department of Public Health, University of Zambia School of Medicine, Lusaka, Zambia; Department of Paediatrics, West Middlesex University Hospital NHS trust, Isleworth, Twickenham, London, UK

**Keywords:** *Panton-Valentine Leukocidin*, *Staphylococcus aureus*, *Methicilin Resistant Staphylococcal aureus*, Community acquired, Brain abscess

## Abstract

**Background:**

Brain abscess are uncommon childhood infection. Brain abscess caused by Panton-Valentine Leukocidin positive Community acquired Methicillin Resistant Staphylococcal aureus have never been reported in the United Kingdom.

**Case presentation:**

We report a case of a previously well 11-month old boy of Indian origin who developed a parietal lobe abscess from PVL positive CA-MRSA.

**Conclusion:**

This case is one of the few described cases of brain abscess caused by PVL CA-MRSA in children. The unusual (insidious) presentation, the absence of a clear staphylococcal focus and the unexpected finding of a CA-MRSA in this patient highlight the challenges of managing such cases in clinical settings and the potential future risk to public health.

## Background

Brain abscess are an uncommon childhood infection [1, 2], but remain a serious and life-threatening disease despite advances in diagnosis and management [1]. Early diagnosis based on clinical signs and symptoms, and appropriate investigation is associated with a better prognosis. The availability of modern neuro-imaging techniques has improved diagnosis of many brain pathologies including brain abscess [1, 3]. The other important factor responsible for improvement in outcomes in recent time has been access to potent antibiotic for most of the organisms causing brain abscesses [1, 4].

The most common causes of brain abscess in children are aerobic and anaerobic streptococci (60 to70% of cases) followed by gram-negative anaerobic bacilli (20 to 40%). Enterobacteriaceae make up 20 to 30%. *Staphylococcus aureus* comprise less than 15% [1]. Community acquired Methicilin Resistant Staphylococcal aureus (CA-MRSA) brain abscess is very rare in children with less than 5 cases reported in literature [1, 5]. Panton-Valentine Leukocidin (PVL) is a toxic substance produced by some strains of *Staphylococcus aureus* which is associated with an increased ability to cause disease [6–8]. We report a case of a previously well 11-month old Indian boy who developed a parietal lobe abscess from PVL positive CA-MRSA.

## Case presentation

A previously fit and well 11-month old Indian boy, initially presented to his local hospital with a 2 week history of intermittent fevers, increasing irritability and anorexia. There was a 24-hour history of diarrhea and vomiting prior to admission. Additionally there was a history of foreign travel to India for 6-months, having returned to the United Kingdom (UK) one-month prior to this presentation. Apart from being lethargic when febrile, he was otherwise well, eating and drinking, and had no neurological deficits. Urine collected at that time grew *E.coli*. He was kept for observation and commenced on oral Co-amoxiclav before being discharged to complete a 7-day course. At this time his C-reactive protein (CRP) was 24 mg/dl(normal range: 0–1.0), White Cell Count (WCC) 25x10^3^ (Normal range 4.5-10^3))^(neutrophils 14) and blood cultures were negative.

Two weeks later, he re-presented with symptoms of persistent fever, irritability, diarrohea & vomiting and photophobia, he was noted to have lost about 1 kg of his weight. Repeat CRP was 83 with a serum WCC of 30. Examination revealed a drowsy, listless ill child that was photophobic. There was no neck stiffness or skin rash. There were no abnormal neurological signs.

Lumbar puncture revealed a cerebral spinal fluid (CSF) WCC of 328(90% polymorphs), CSF protein was 1.2 g/l and glucose of 1.7 mmol/l compared to 7 mmol/l in the blood. No organisms were seen. A provisional diagnosis of partially treated meningitis was made and the child was commenced on ceftriaxone. Quadruple anti-tuberculous medication (rifampicin, ethambutol, isoniazid and pyrazinamide) was also instigated in view of the recent foreign travel.

**Figure 1:**
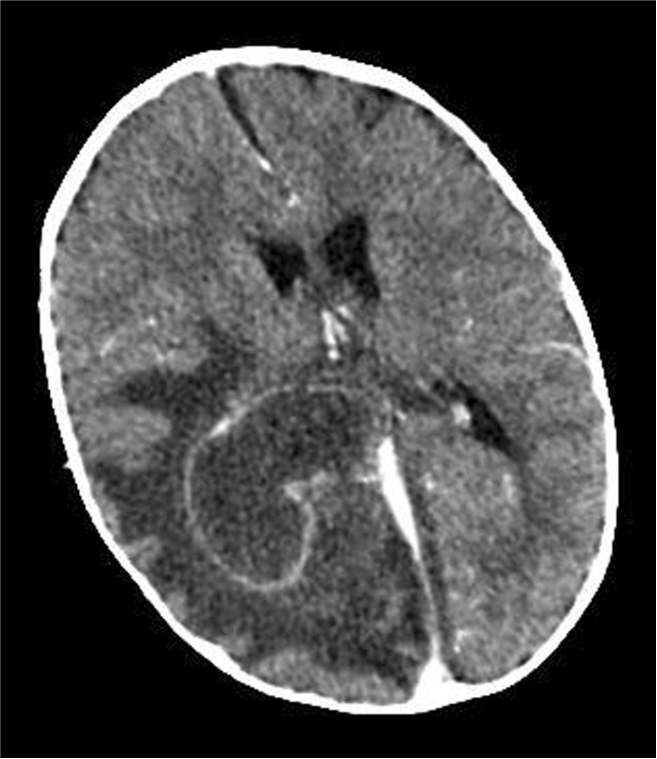
**Pre-operative contrast enhanced CT scan.**

Because of persisting drowsiness a Computerized Tomography (CT) brain was performed. This revealed a large lobulated low attenuation mass centered over the right occipito-parietal region with extensive surrounding vasogenic oedema consistent with an intracerebral abscess. The scan also revealed moderate midline shift and a low density left parafalcine subdural effusion Figure 1.

The child was transferred for urgent neurosurgical treatment. Forty-eight mls of pus were aspirated via an image guided needle aspiration of the right parietal lesion, in which gram positive cocci were seen. Vancomycin, amikacin and metronidazole were started, with the cefotaxime and rifampicin. Post-operatively the child’s clinical condition improved.

PVL CA-MRSA was cultured from the brain abscess sample. Antibiotic sensitivity testing showed that the bacteria was only resistant to Flucloxacillin, ciprofloxacin and trimethoprim, sensitive to gentamicin, Amikacin, Erythromycin, Fucidin, Tetracycline, Linezolid, Vancomycin and Rifampicin. The CSF and blood cultures from the local hospital also grew MRSA. With the quantiferon test negative for Tuberculosis, antitubercuous medication was discontinued and the antibiotic regime was rationalized to intravenous Rifampicin, Vancomycin and Linezolid. The isolate was referred to the Staphylococcal Reference Laboratory, Colindale, Public Health England and shown to be PVL gene positive. Typing showed it was spa type 021 with repeat succession 15-12-16-02-16-02-25-17-24 which is associated with PVL-MRSA belonging to MLST clonal complex 30 (so-called South-West Pacific clone) reported in the SWP region. The nose and throat MRSA screen, and subsequent surface screens, were all negative.

**Figure 2:**
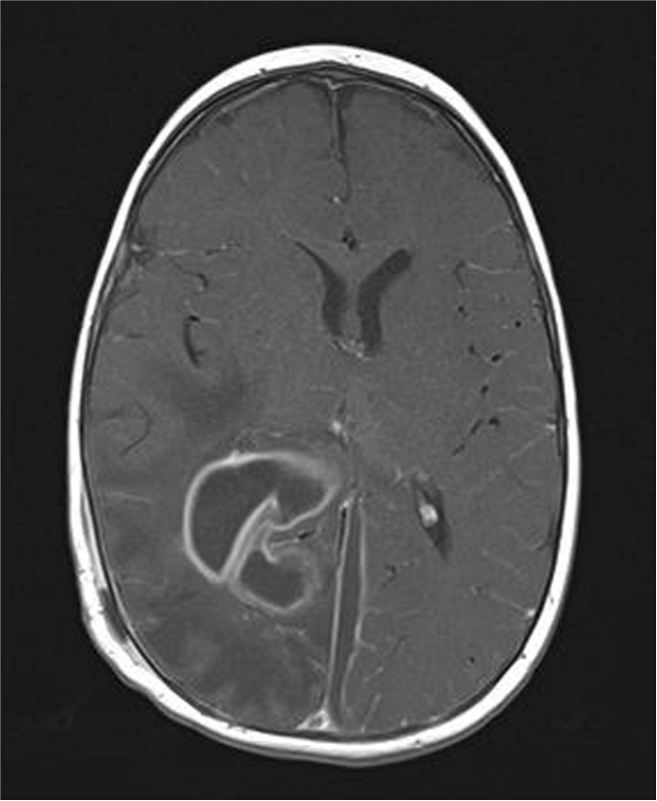
**Initial post-op magnetic resonance imaging.**

An MRI brain, performed 2 days after the needle aspiration (Figure 2), showed considerable reduction in the size of the parieto-occipital abscess with persistence of the parafalcine collection. Given the clinical improvement and the established microbiological diagnosis no further surgery was performed and antibiotics were continued.

**Figure 3:**
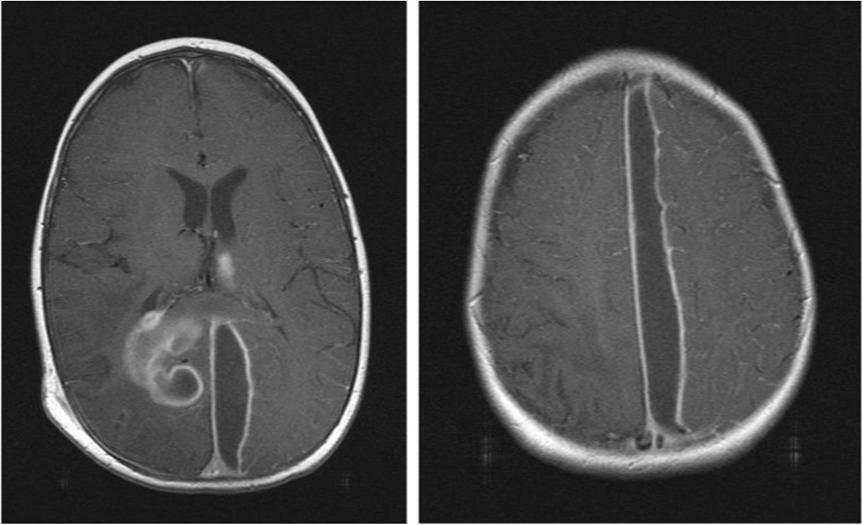
**Magnetic resonance imaging 1 week after admission.**

**Figure 4:**
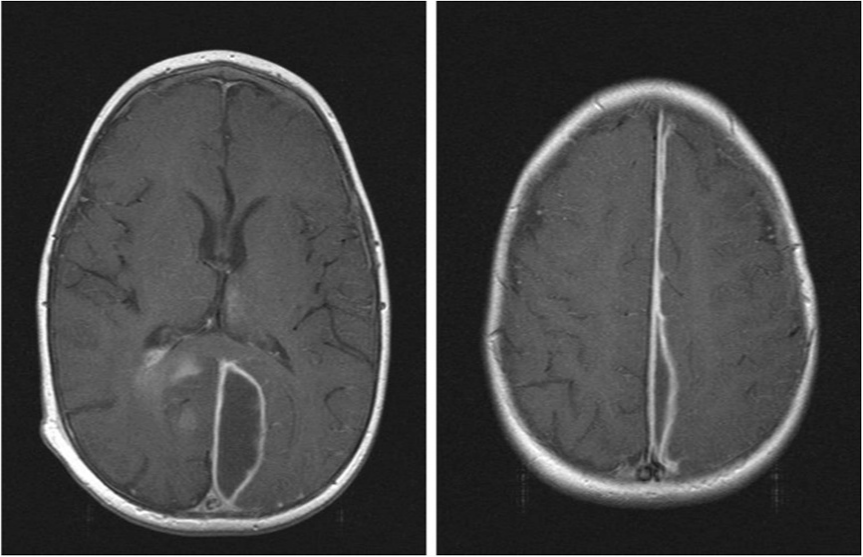
**Magnetic resonance 2 weeks after admission & 1 week post 2nd operation.**

However one-week after surgery MRI demonstrated progression of the parafalcine collection (Figure 3) necessitating a second neurosurgical procedure. The parafalcine collection was drained through a right frontal craniotomy Figure 4. The pus aspirated was still culture positive for MRSA after extended broth enrichment.

**Figure 5:**
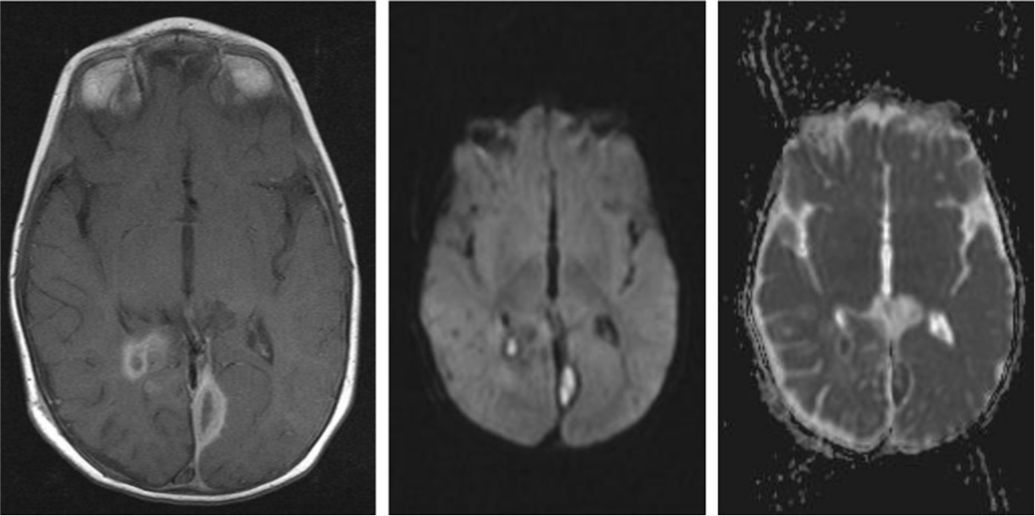
**Magnetic Resonance Imaging 6 weeks after admission.**

**Figure 6:**
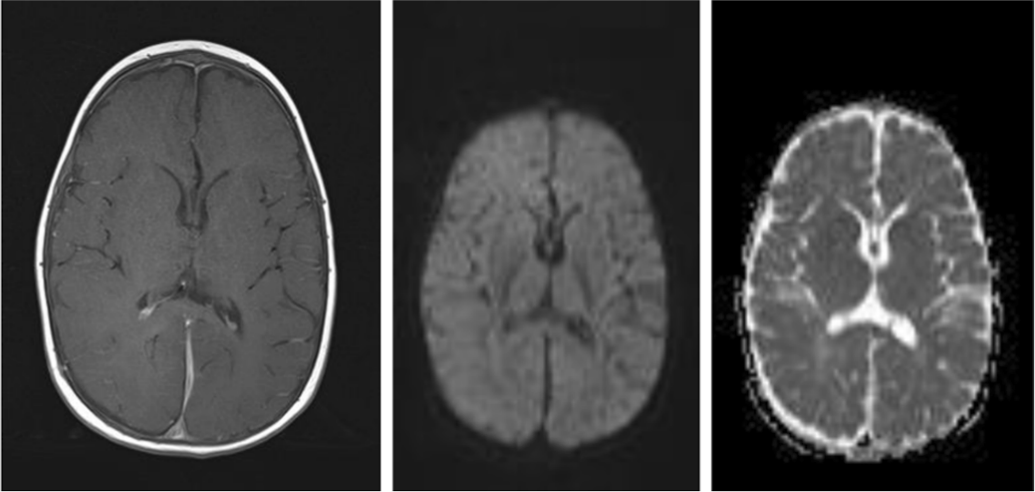
**Magnetic Resonance Imaging 12 weeks after admission.**

After 6 weeks of antibiotic therapy Magnetic Resonance Imaging (MRI) showed almost complete resolution of the abscess and empyema. The diffusion sequences showed persistent active infection (Figure 5) and as such the antibiotics were continued for a further 2 weeks and then stopped. A final MRI (Figure 6) after 12 weeks (1 month off antibiotics) revealed resolution of the right parieto-occipital abscess and left parafalcine collection without any diffusion MRI evidence of active infection. At this time the child was very well, had no neurological deficits and had been afebrile with a CRP <5 for a month. The patient has remained well after 10 months of follow-up.

## Discussion

*Staphylococcus. aureus* is a major human pathogen, causing a wide range of disease in humans from simple skin infection to overwhelming sepsis and death. Serious infections occur most often in the first year of life and above the age of 65 years [7, 8]. Whilst *S. aureus* is known to cause infection of the central nervous system, it is most frequently a post-operative or trauma related pathogen, and it is a very rare pathogen in community-onset meningitis or brain abscess [1, 2, 9]. The organism has a wide range of virulence factors, including the Panton-Valentine Leukocidin, the possession of which is reported to be associated especially with skin infection and necrotising pneumonia [6, 7, 10, 11], although its importance is not universally excepted [8]. In addition to the multiple virulence factors, *S. aureus* may be resistant to the commonly used class of antibiotics including meticillin (hence called MRSA). While MRSA is classically associated with health care associated (HCA) strains, it more recently has been detected in strains arising in the community (Community associated, CA-MRSA) [7, 8]. Unlike HCA MRSA, CA-MRSA is usually PVL positive [7, 8]. Rapid spread of CA-MRSA has been recognised in certain areas or communities e.g. a North American clone in USA, designated USA300 or a south west Pacific strain, designated the SWP clone. The SWP clone is a rare cause of infection in the UK, with one outbreak only reported [7]. Brain abscess caused by PVL CA-MRSA are very rare and only two cases have been described in adults [12, 13]. To our knowledge this is the first brain abscess caused by PVL CA-MRSA in an infant.

Intracranial microbial infection in children may occur by direct extension from the middle ear, paranasal sinuses or indirectly via the haematogenous route from dental or cardiac origin. However, findings from our case did not clearly point to these well known sources of brain infection. There was no evidence of ear or nasal infection and the patient had no history of skull trauma, surgery or osteomyelitis. This is in contrast to other reported cases of PVL CA-MRSA brain abscess in adults which have shown clearly source of brain inoculation [6, 10, 12, 13]. Additionally, there was no evidence of a primary cutaneous or other S. aureus focus.

The patient had been in India for 6 months before presenting and is most likely to have acquired the SWP CA-MRSA clone while abroad travelling. Although community acquired MRSA is prevalent in the Indian subcontinent due to widespread antibiotic use, there is no routine policy in the UK to evaluate children for MRSA after foreign travel to endemic countries [14, 15], as there is for other multiple antibiotic resistant organisms. In cases such as ours, tuberculosis could be considered more likely in such children and MRSA is not one of the major public health risk for many children who frequently travel between UK and India. This case presented with very none specific symptoms and was treated together with all other children in the hospital up to 3 weeks before the diagnosis was made. Most general paediatric units do not have a policy to screen children for MRSA, however, this case demonstrates it is clearly important to have a high index of suspicion when dealing with children with history of international travel who apparently have no other risk factors.

The CT-scan was not performed until approximately 3 weeks after initial symptoms. This case demonstrates that life threatening intracranial sepsis with mass lesions can develop insidiously in the absence of focal neurological deficits or significantly impaired level of consciousness. More evidence is required to guide policy and management of similar cases.

Immunological evaluations including HIV tests showed that the child had normal immunity and the parents reported no health issues. In line with national policy for management of PVL-MRSA this child and the family were referred to Public Health for further management [8]. Topical decolonisation was given, although this child had not screened positive on surface swabs. In view of the intracranial location of the infection (with poor vancomycin penetration), and PVL positivity (experience with necrotising pneumonia and PVL infection suggests use of drugs that may reduce toxin production should be used), therapy was initially continued with vancomycin, linezolid and rifampicin. As the second aspirate, after 8 days of good anti-MRSA therapy was still culture positive, he was further continued on all 3 agents until the 6 week review.

There are a number of neurosurgical strategies that are recommended for the treatment of brain abscess or empyema ranging from simple aspiration through to craniotomy and excision of the abscess. The guiding principles of management are to establish an accurate microbiological diagnosis and to reduce mass effect. As demonstrated in this case, it is not uncommon to require more than one surgical intervention in the course of treatment. There is some evidence to suggest that more minimalist procedures are equally effective in treatment of intracranial sepsis. Furthermore some have begun to suggest that antibiotic courses could also be reduced, but we did not do so here, extending the treatment longer because of the slow resolution.

## Conclusion

This case is one of the few described cases of brain abscess caused by PVL CA-MRSA in children. There was no evidence of ear or nasal infection and the patient had no history of skull trauma, surgery or osteomyelitis. The unusual (insidious) presentation, the absence of a clear staphylococcal focus and the unexpected finding of a CA-MRSA in this patient highlight the challenges of managing such cases in clinical settings and the potential future risk to public health from these virulent pathogens associated with travel.

## Consent

Written informed consent was obtained from the patient’s parents for publication of this Case report and any accompanying images. A copy of the written consent is available for review by the Editor of this journal.

## Abbreviations

PVL: *Panton-Valentine Leukocidin*
MRSA: *Methicilin Resistant Staphylococcal aureus (MRSA)*
CA: Community acquired
CSF: *Cerebral spinal fluid*
CRP: C-reactive protein
WCC: White cell count
SWP: South West Pacific
HCA: Health care associated.
